# Rubella Eradication: Not Yet Accomplished, but Entirely Feasible

**DOI:** 10.1093/infdis/jiaa530

**Published:** 2021-09-30

**Authors:** Stanley A Plotkin

**Affiliations:** Emeritus Professor of Pediatrics, University of Pennsylvania, Vaxconsult, Doylestown, Pennsylvania, USA

**Keywords:** rubella, measles, congenital, eradication

## Abstract

Rubella virus is the most teratogenic virus known to science and is capable of causing large epidemics. The RA 27/3 rubella vaccine, usually combined with measles vaccine, has eliminated rubella and congenital rubella syndrome from much of the world, notably from the Western Hemisphere. Except in immunosuppressed individuals, it is remarkably safe. Together with rubella vaccine strains used in China and Japan, eradication of the rubella virus is possible, indeed more feasible than eradication of measles or mumps.

In 1993, the International Task Force for Disease Eradication published its recommendations of the list of diseases that could and could not be eradicable at that time [1]. Rubella and mumps were on the list of diseases that could be eradicated now; measles was not because of its infectiousness and vaccine ineffectiveness in infants <9 months of age. However, because of the significant morbidity and mortality of measles, the Pan American Health Organization (PAHO) established a goal in 1994 to eliminate measles by 2000. Subsequently, all of the other World Health Organization (WHO) regions established a goal of measles elimination 2020 or before. In 2016, PAHO succeeded in reaching measles elimination; however, that goal was lost in 2018 owing to reestablishment of endemic measles in Venezuela [2]. In 2003, PAHO established a rubella elimination goal, which it succeeded in reaching in 2009 and verified in 2015 and has maintained [3]. Currently 3 other WHO regions (Europe, South-East Asia, and Western Pacific) have rubella elimination goals. No region has established a mumps elimination goal, as the mumps vaccine is only partially effective [4]. Measles is justifiably very much in the news today, owing to recrudescence of the disease caused by the numbers of unvaccinated children. Owing to the serious and frequent complications of measles, much effort is being devoted to convince parents to vaccinate their children. Nevertheless, those efforts are having uneven results owing to parental resistance in developed countries, but also because of the high infectiousness of the measles virus, which means practically everyone must be immune to stop viral circulation and disease in the unvaccinated population. In 2019, the International Task Force for Disease Eradication (ITFDE) met to discuss the potential for disease eradication for measles and rubella in light of the massive resurgence of measles. Despite measles outbreaks, the ITFDE continues to firmly consider that eradication of both measles and rubella is technically feasible and that both should be eradicated [5].

The current rubella vaccine was conceived and developed in the shadow of measles vaccination. Attenuated measles vaccine had just been developed, but experience with a candidate inactivated measles vaccine had demonstrated enhancement of disease and unreliable efficacy. The rubella epidemic of 1963–1965 in Europe and North America stimulated the development of several attenuated rubella strains. During the late 1960s, accumulated data on safety and efficacy showed that the RA27/3 rubella vaccine that is produced in human fetal diploid cells had superior immunogenicity, leading to its production and adoption for immunization of infants and women of childbearing age. The combination of rubella with measles and mumps vaccine by Maurice Hilleman gradually led to widespread adoption of measles-mumps-rubella (MMR) as a universal childhood vaccine in developed countries. Whereas measles vaccine was rapidly adopted in many low-income countries, it was adoption of rubella vaccination by PAHO that led to widespread use of the rubella vaccine. The later adoption by WHO of elimination of measles and rubella as a public health goal, together with the support of Gavi the Vaccine Alliance to provide measles-rubella (MR) vaccine to low-income countries, has resulted in elimination of rubella from large parts of the world.

Because of the high effectiveness of the measles and rubella components, elimination and subsequent eradication of those 2 diseases has become a public health goal, owing to increasing confidence in those vaccines. However, strategies to achieve those goals have evolved with time.

Rubella was historically viewed as a variant of measles or scarlet fever. After a rubella epidemic in Australia in the early 1940s, Australian ophthalmologist Sir Norman Gregg noticed the occurrence of congenital cataracts among infants whose mothers had reported rubella during early pregnancy, and he first described the congenital rubella syndrome (CRS). In late 1962, 2 separate groups of scientists in the United States isolated the rubella virus. During 1962–1963, a rubella epidemic broke out in Europe, spreading to the United States in 1964–1965. That rubella epidemic resulted in >30 000 infections during pregnancies. CRS occurred in approximately 20 000 infants born alive, including >11 000 infants who were deaf, >3500 infants who were blind, and almost 2000 infants who were mentally retarded. The medical cost of this epidemic exceeded $1.5 billion in 1965 dollars. With the isolation of the virus and the availability of diagnostic tests, the epidemic led to the recognition of an expanded CRS, adding hepatitis, splenomegaly, thrombocytopenia, encephalitis, and mental retardation to the already described triad of deafness, cataracts, and heart disease. The devastation of the epidemic and the ability to grow the rubella virus contributed to the urgency to develop a vaccine. Between 1965 and 1967, several attenuated rubella strains were developed and reached clinical trials. In 1969 and 1970, rubella vaccines entered into commercial use in Europe and North America. Eventually the RA27/3 rubella strain grown in human fetal diploid cell strains became the sole vaccine in use, except in Japan and China [6].

Thus, vaccination against rubella has been conducted since the late 1960s with little fanfare. Remarkable progress has been made in the introduction of rubella-containing vaccine (RCV) in developing countries, thus, reducing global inequity in its use and reducing the numbers of reported cases of rubella and of CRS, although approximately 105 000 infants are still born each year with preventable CRS [5].

The incubation period of rubella is at least 14 days, which is important, as it permits anamnestic responses to vaccination to be stimulated by exposure to the virus and thus to prevent disease even if antibody titers have declined to low levels years after vaccination. This probably explains why outbreaks have failed to occur in immunized populations, but it also makes definition of a correlate of protection more difficult. In general, neutralizing titers of ≥1:8 and enzyme-linked immunosorbent assay titers of ≥10 IU are considered to be protective. Although reinfection has been observed in vaccinees, transmission of rubella virus from vaccinated pregnant women to their fetuses has been rare. Moreover, although 1 dose of rubella vaccine has been remarkably effective, its combination with measles and mumps vaccines has resulted in the broad use of 2 doses.

## HISTORY OF RUBELLA VACCINATION PROGRAM STRATEGIES

In 1969, several rubella vaccines were licensed; however, there was no consensus internationally as to which groups to target for vaccination. The 2 basic approaches were (1) universal vaccination (targeting children) or (2) selective vaccination of susceptible adolescent girls and women of childbearing age. The universal approach was designed to interrupt transmission of the virus by vaccinating the reservoir of infection in children, thus reducing the overall risk of infection in adults and providing indirect protection of unvaccinated, postpubertal women. The selective vaccination of women directly protects women at risk of being infected when pregnant, but allows virus to circulate and thus boost vaccine-induced immunity in the population. Countries using the selective vaccination approach were unsuccessful because of the inability to immunize a sufficient proportion of the female population. With this approach, large-scale epidemics continued to occur, and the incidence of CRS did not decline significantly. In addition, countries that set goals to eliminate rubella could not achieve it through the selective vaccination approach. After 20 years of experience, it is clear that the integration of the 2 approaches, vaccination of children and adults, is necessary for maximum control. In 2011, after reviewing the successes with RCVs through mass vaccination in the PAHO and other regions, the WHO rubella vaccine recommendations were updated [7]. Countries that had not introduced rubella vaccination were recommended to take the opportunity offered by measles accelerated control and elimination to introduce rubella vaccine. There are 2 approaches to the introduction of rubella vaccine: (1) universal vaccination, or (2) selective vaccination of women and adolescent girls. The preferred approach for introduction of rubella vaccination is to begin with a campaign targeting a wide range of ages (usually 9 months to 15 years) of both males and females together with an immediate introduction of MR/MMR vaccine into the routine vaccination of infants. In 2020, the WHO updated its guidance for rubella vaccine introduction to recommend that routine vaccination of infants should be the primary focus, with an introductory wide age range campaign (usually 9 months to 14 years).

## HISTORY AND STATUS OF RUBELLA INTRODUCTION AND PROGRESS TOWARD ELIMINATION BY REGION

Unlike measles regional elimination goals that are established in all 6 WHO regions, only 4 WHO regions have established rubella elimination goals. The WHO regions are at different stages in their progression toward elimination. In 2000, only 99 countries (51%) had introduced RCV in their national childhood programs [3]. However, as of January 2020, 173 countries (89%) have introduced rubella vaccine into their national programs. There has been a sharp decline in the number of rubella cases, from 670 894 in 2000 to 26 006 in 2018 [8]. Nevertheless, due to large epidemics in China and Japan, the number of cases increased to >30 000 cases in 2019.

### Region of the Americas

In 2015, the WHO Region of the Americas (AMR) verified elimination of rubella in all 35 countries and territories. Thus, AMR is the first and only WHO region to achieve regional rubella elimination. In 1997, as part of a regional initiative for rubella control and CRS prevention, PAHO developed a rubella and CRS control strategy that included introduction of a RCV into routine childhood immunization programs, ensuring rubella vaccination of women of childbearing age to reduce the number of susceptibles. In addition, 2 specific vaccination strategies for accelerated rubella control and CRS prevention were developed. Countries wishing to prevent and control CRS promptly were advised to conduct a 1-time mass campaign to vaccinate all females 5–39 years of age with measles and rubella–containing vaccine, and countries wishing to prevent and control both rubella and CRS promptly were advised to carry out a 1-time mass campaign to vaccinate males and females 5–39 years of age with measles and rubella–containing vaccines [9]. With Caribbean subregional countries reaching their rubella elimination goal and with additional progress in the rest of the region, in 2003 PAHO adopted a resolution calling for rubella and CRS elimination in the Americas by the year 2010. To accomplish this goal, PAHO advanced a rubella and CRS elimination strategy including introduction of RCVs into routine vaccination programs, leading to high immunization coverage, interruption of rubella transmission through mass vaccination of adolescents and adults, and strengthened surveillance for rubella and CRS. The speed-up campaign involved vaccinating males and females up to 40 years of age. However, 3 countries chose to vaccinate only adolescent and adult females, leading to subsequent outbreaks mainly among adolescent and adult males. Moreover, infants with CRS continued to be born. Each of these 3 countries conducted additional campaigns including males that had been excluded in the first speed-up campaigns. The last endemic rubella case in the PAHO region occurred in 2009 [10]. In 2015, the Americas were verified to be free of endemic rubella and CRS. Up to now, the region of the Americas has sustained elimination of rubella and CRS [3].

### European Region

The WHO European Region (EUR) is comprised of 53 member states, including Western and Eastern Europe, Russia, and the Newly Independent States. In the 1970s, many of the countries chose to introduce RCV using the selective targeting of women and adolescent girls. However, in the 1980s, many countries chose to add vaccination into routine childhood programs, resulting in a combined childhood and female vaccination strategy. Starting in 2000, many of the countries chose to introduce RCV through a wide-age childhood campaign followed by introduction of RCV into the routine pediatric program. Some countries also chose to target adult females through routine and/or campaign vaccination. In 2005, EUR became the second region to establish a rubella elimination goal by 2010 [11]. By 2009, all countries in the region had introduced RCV into their national program. However, in the late 2000s, several countries continued to experience large rubella outbreaks. The 2010 goal was not achieved, so the goal was changed to 2015. In September 2014, all member states reaffirmed their commitments to the goal of measles and rubella elimination as part of the European Vaccine Action Plan 2015–2020 [12]. With progress being made toward achievement of elimination, the rubella case counts decreased sharply from 621 039 cases in 2000, to 798 in 2018. In 2015, 24 (45%) countries had eliminated rubella and 11 (21%) had interrupted transmission. Of the 53 countries, 10 EUR countries have not been verified as eliminating rubella; however, progress is being made to promote acceptance of the importance of vaccines and thus to complete the task in that geographic area.

### Western Pacific Region

In 2012, the Regional Committee of the WHO Western Pacific Region (WPR) agreed to accelerate rubella control; in 2017, it resolved that all countries or areas should aim for rubella elimination as soon as possible. In the 1970s, many countries chose to introduce RCV through the selective targeting of women and adolescent girls. However, in the 1980s and early 1990s, many of those countries introduced vaccination into routine programs, resulting in a combined childhood and selective female vaccination strategy. Starting in 2009, countries introduced RCV through a wide-age childhood campaign followed by introduction in to the routine program. By 2015, all 36 countries had introduced RCV. Coverage with a first dose of RCV increased from 11% in 2000 to 96% in 2019. Whereas reported rubella incidence increased from 35.5 to 71.3 cases per million population among reporting countries during 2000–2008, it decreased to 2.1 in 2017, but then increased to 18.4 in 2019 because of outbreaks in China and Japan. As of 2019, 5 countries and areas (Australia, Brunei Darassalam, Macao [China], New Zealand, and Republic of Korea) have been verified as eliminating rubella [13].

### South-East Asia Region

In 2013, the 66th session of the Regional Committee of the WHO South-East Asia Region (SEAR) adopted the goal of elimination of measles and control of rubella and CRS by 2020. By the end of 2016, 8 (73%) countries had introduced RCV. By the end of 2019, India, Indonesia, and North Korea, 3 countries that account for 84% of infants living in the region, had introduced RCV into their national programs. However, with improvement in MR surveillance, the case counts have increased from 1165 in 2000 to 4533 in 2018. In 2018, Bangladesh, Bhutan, Maldives, Nepal, Sri Lanka, and Timor-Leste were verified to have controlled rubella/CRS [3]. Because of the significant progress of rubella control, the regional committee voted in 2019 to establish a rubella elimination goal by 2023. In 2020, 2 countries (Maldives and Sri Lanka) have been verified to achieve rubella elimination.

### Eastern Mediterranean Region

There are 22 countries and 1 area in the WHO Eastern Mediterranean Region (EMR). This region currently does not have regional rubella elimination goals; however, 13 countries have national rubella and CRS goals. Currently, there are 16 countries and 1 geographical area that have introduced rubella vaccination. Rubella incidence in EMR is associated with the 5 countries (Afghanistan, Pakistan, Djibouti, Sudan, and Somalia) that have not introduced rubella vaccine into their programs. With the improvement in MR surveillance, the rubella case counts have slightly decreased from 3122 in 2000 to 1622 in 2019. In 2019, 3 countries were verified as achieving rubella elimination: Iran, Bahrain, and Oman.

### African Region

The WHO African Region (AFR) consists of 47 countries. At this time, AFR does not have a regional rubella control or elimination goal. Prior to 2010, Cape Verde, Mauritius, and the Seychelles introduced MR vaccine. Between 2012 and 2020, 28 additional countries introduced MR vaccine through mass campaigns targeting children between 9 months and 14 years of age, prior to introduction of vaccine in their childhood immunization programs. The remaining 16 countries are mainly in Central and Western Africa. With the improvement of MR surveillance, rubella cases are being identified. The rubella case counts have increased from 865 in 2000 to 11 787 in 2018. The countries introducing MR after 2011 utilized similar strategies to the first successful countries, and although no country has achieved rubella elimination, progress has been reported [14].

## GLOBAL ELIMINATION STATUS

Rubella elimination is defined as the interruption of endemic rubella virus transmission for at least 12 months. When interruption of transmission is sustained for 36 months, an independent regional commission verifies countries as having eliminated rubella.

Rubella and CRS regional elimination goals have been established by AMR, EUR, SEAR, and WPR. AFR and EMR do not yet have an elimination goal. AMR was verified to have eliminated rubella and CRS in 2015. Verification commissions in EMR, EUR, SEAR, and WPR assess rubella elimination status country by country. The elimination of endemic rubella has been verified in 83 countries: 3 of 23 (13%) in EMR, 39 of 53 (74%) in EUR, 2 of 11 (18%) in SEAR, 4 of 27 (15%) in WPR, and 35 of 35 (100%) in AMR.

## DISCUSSION

**Figure 1.**
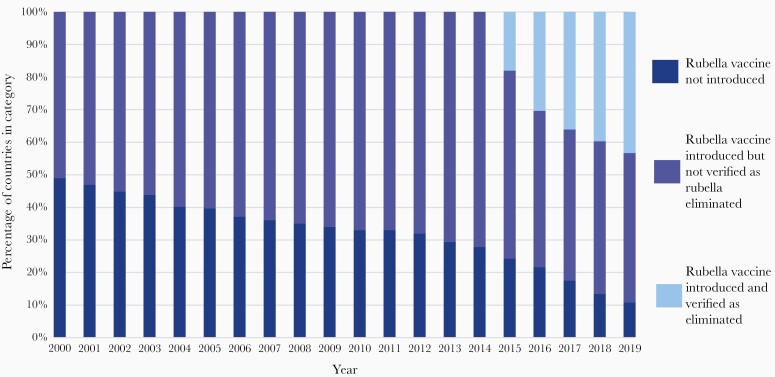
Global progress of countries that have introduced rubella-containing vaccine and the number of countries that have eliminated rubella transmission, 2000–2019. Source: World Health Organization.

**Figure 2.**
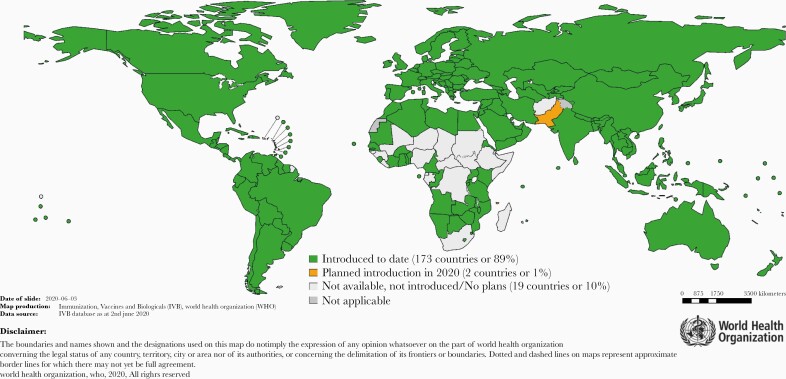
Countries with rubella vaccine in the national immunization program, and planned introductions in 2020. WHO data and on-line information are in the public domain.

Since the introduction of rubella vaccine in 1969, significant progress has been made in eliminating rubella globally. As of 8 July 2020, 84 of 195 (43%) countries have eliminated rubella (Figures 1 and 2), 4 regions have elimination goals, and 173 of 195 (89%) countries have introduced rubella in their program.

Even with these successes, still it is estimated that >100 000 infants are born with CRS annually. The lifetime costs for medical care of a child with CRS are estimated to range from $11 000 in low-income countries to approximately $1 000 000 in high-income countries [15]. However, with the increased use of rubella vaccine, 131 000 deaths and 12.5 million disability-adjusted life-years due to CRS may be prevented from 2001 to 2030 [16].

As highlighted in this article, epidemiological, laboratory, and clinical data were used to modify the rubella vaccine strategy. Experience with various strategies was reviewed and lessons learned were applied. The WHO updated the recommended strategy in 2020 its latest rubella vaccine position paper. No longer is it recommended to vaccinate women of reproductive age only, but rather such vaccination must be combined with routine childhood vaccination. It is recommended that all countries introducing RCV into their national program should conduct an introductory wide-age campaign.

In 2009, an ad hoc Global Measles Advisory Group was convened by WHO, leading to a global technical consultation to assess the feasibility of measles eradication in 2010 [17]. On the basis of this review, the WHO Strategic Advisory Group of Experts on Immunization (SAGE) concluded that measles could and should be eradicated [18]. In 2015, the ITFDE proposed that rubella eradication was also feasible and encouraged discussion on the feasibility and potential timing [19]. At the October 2019 WHO SAGE meeting, the feasibility of measles and rubella eradication was reviewed [20]. It was noted that achievement and maintenance of the elimination threshold for rubella is more likely and would occur earlier than elimination of measles. However, SAGE concluded that while accelerating toward measles and rubella elimination is urgent, eradication is not a realistic outcome in the short or medium term. Establishment of a goal and target date will be considered only when substantial, measurable progress has been made.

Nevertheless, wherever rubella vaccine has been employed, the rash disease and its congenital infection seem to disappear. In contrast, measles outbreaks continue in Europe and the United States, as vaccination coverage is low in certain areas. Why the difference? First, rubella is less infectious than measles, with the reproductive number (Ro) being about 7 for the former but about 12 for the latter. Second, immunity to measles may be less robust, in that vaccinees with lower titers of antibody often show booster responses indicating subclinical infections [20]. Antibody titers after rubella vaccination may drop to low levels, but resistance to infection appears to persist [21, 22]. Also, the incubation period of rubella (14–23 days) is longer on average than that of measles (10–21 days), which means that there is more time for an anamnestic response in vaccinees to suppress rubella virus replication after exposure than to suppress measles virus replication [6].

Despite great progress in rubella elimination, there are concerns. Whereas rubella and CRS have been eliminated from the Americas [23, 24], they persist in parts of Europe resistant to vaccination. Whereas some European countries like the United Kingdom and Spain have been quite successful in eliminating rubella [25, 26], other countries like France and Italy have had difficulty [27–29].

In Asia the situation is not fully elucidated, but CRS is frequently reported in India [21, 22, 30, 31]. Seropositivity in Chinese women is incomplete [32, 33], but vaccination has been said to be reducing rubella cases [34]. On the other hand, incomplete vaccine coverage in men in Japan has resulted in persistent rubella outbreaks [35–37]. Australia is on track for rubella elimination [29, 38], but the situation in the Pacific Islands is uncertain, as shown by a cluster of CRS cases in the Solomon Islands [39]. In Africa, recent introduction of RCV has demonstrated an impact on the incidence of rubella [14], but the impact on CRS is uncertain [40–46].

The overall reported safety of rubella vaccine continues to be good in normal women and children, including pregnant women (in whom the vaccine is nevertheless contraindicated). Remarkably, although contraindicated, the RA27/3 vaccine strain in pregnant women does not cause CRS, despite the occasional transmission of virus to the fetus [47]. As a live virus vaccine, it is contraindicated in subjects with impaired immunity, which could allow the virus to persist despite vaccination [48].

Not surprisingly, given the frequently serious and even fatal disease caused by measles virus, greater attention is being directed toward measles eradication. However, it may be far easier to eradicate rubella through vaccination. Considering the difficulties now apparent after >30 years of attempts to eradicate polio, and the setbacks in measles eradication owing to parental resistance to vaccination or to limited access to vaccination, perhaps it is time to refocus goals toward the elimination of rubella, in order to give the world a more feasible victory. The general use of MR or MMR combinations rather than measles vaccine alone should enable the elimination of rubella. Factors that make rubella eradication easier than measles eradication include; good persistence of rubella antibodies [49] and the apparent lower rate of breakthrough infections, possibly because the longer incubation period of rubella allows anamnestic immune responses to protect. Nevertheless, about 100 000 CRS cases are estimated to occur annually in 92 low- and middle-income countries [16], and 77% of countries now report CRS.

The need now is to make rubella eradication a goal as part of the efforts to control measles [23, 50, 51]. A public health goal of rubella eradication should be widely announced and efforts made to take advantage of its feasibility in the context of the efforts to control measles. The choice of disease targets for eradication is not simple. The difficulty in eliminating polio has raised concerns about feasibility. Eradication of measles may also be difficult because of its high infectivity. Therefore, consideration should be given to making rubella eradication the most immediate target.

## Supplementary Material

jiaa530_suppl_Supplementary-Material

## Notes

***Acknowledgments.*** Much of the information in this article was furnished by Dr Susan Reef, to whom I am grateful. Unfortunately, CDC rules about publication authorship could not be satisfied in time to permit her to be a coauthor.

***Supplement sponsorship.*** This supplement is sponsored by the Bill and Melinda Gates Foundation.

***Potential conflicts of interest*.** S. A. P. consults for vaccine manufacturers, including those that make rubella vaccine.

S. A. P. has submitted the ICMJE Form for Disclosure of Potential Conflicts of Interest. Conflicts that the editors consider relevant to the content of the manuscript have been disclosed.
