# Supplementary material for: Rubella Eradication: Not Yet Accomplished, but Entirely Feasible
Source: J Infect Dis. 2021 Sep 30;224(Suppl 4):S360–6. doi: 10.1093/infdis/jiaa530 (PMC8482023; doi:10.1093/infdis/jiaa530)
Supplement: jiaa530_suppl_Supplementary-Material [file jiaa530_suppl_supplementary-material.docx]

REFERENCES

1. Recommendations of the International Task Force for Disease Eradication. MMWR Recomm Rep **1993**; 42(RR-16):1–38.

2. Patel MK, Dumolard L, Nedelec Y, et al. Progress toward regional measles elimination—worldwide, 2000–2018. MMWR Morb Mortal Wkly Rep **2019**; 68:1105–11.

3. Grant GB, Desai S, Dumolard L, Kretsinger K, Reef SE. Progress toward rubella and congenital rubella syndrome control and elimination—worldwide, 2000–2018. MMWR Morb Mortal Wkly Rep **2019**; 68:855–9.

4. Plotkin SA. Mumps: a pain in the neck. J Pediatric Infect Dis Soc **2018**;7:91–2.

5. World Health Organization. Report of the thirtieth meeting of the International Task Force for disease eradication, 22 October 2019. Wkly Epidemiol Rec **2020**; 95:61–8.

6. Reef S. Chapter 53: Rubella vaccines. In: Plotkin’s vaccines. 7th ed. Philadelphia, PA: Elsevier, **2017**:970–1000.

7. World Health Organization. Rubella vaccines: WHO position paper. Wkly Epidemiol Rec **2011**; 86:301–16.

8. World Health Organization. Rubella vaccines: WHO position paper. Wkly Epidemiol Rec **2020**; 95:301–24.

9. Castillo-Solórzano C, Carrasco P, Tambini G, Reef S, Brana M, de Quadros CA. New horizons in the control of rubella and prevention of congenital rubella syndrome in the Americas. J Infect Dis **2003**; 187:S146–52.

10. Castillo-Solórzano C, Marsigli C, Bravo-Alcántara P, et al. Elimination of rubella and congenital rubella syndrome in the Americas. J Infect Dis **2011**; 204:S571–8.

11. Zimmerman LA, Muscat M, Jankovic D, et al. Status of rubella and congenital rubella syndrome surveillance, 2005–2009, the World Health Organization European Region. J Infect Dis **2011**; 204:S381–8.

12. Datta SS, O’Connor PM, Jankovic D, et al. Progress and challenges in measles and rubella elimination in the WHO European Region. Vaccine **2018**; 36:5408–15.

13. Knapp JK, Mariano KM, Pastore R, et al. Progress toward rubella elimination—Western Pacific Region, 2000–2019. MMWR Morb Mortal Wkly Rep **2020**; 69:744–50.

14. Luce R, Masresha BG, Katsande R, Fall A, Shibeshi ME. The impact of recent rubella vaccine introduction in 5 countries in the African Region. J Immunol Sci **2018**; (Suppl):108–12.

15. Thompson KM, Odahowski CL. The costs and valuation of health impacts of measles and rubella risk management policies. Risk Anal **2016**; 36:1357–82.

16. Vynnycky E, Papadopoulos T, Angelis K. The impact of measles-rubella vaccination on the morbidity and mortality from congenital rubella syndrome in 92 countries. Hum Vaccin Immunother **2019**; 15:309–16.

17. World Health Organization. Proceedings of the Global Technical Consultation to assess the feasibility of measles eradication, 28–30 July 2010. J Infect Dis **2011**; 204:S4–13.

18. World Health Organization. Meeting of the Strategic Advisory Group of Experts on Immunization, Nov. 2010. Summary, conclusions and recommendations. Wkly Epidemiol Rec **2011**; 86:1–16.

19. World Health Organization. Meeting of the International Task for Disease Eradication, Nov. 2015. Wkly Epidemiol Rec **2016**; 91:61–70.

20. Plotkin SA. Measles: breakouts and breakthroughs. J Pediatric Infect Dis Soc **2019**; 8:289–90.

21. Dimech W, Mulders MN. A 16-year review of seroprevalence studies on measles and rubella. Vaccine **2016**; 34:4110–8.

22. LeBaron CW, Forghani B, Matter L, et al. Persistence of rubella antibodies after 2 doses of measles-mumps-rubella vaccine. J Infect Dis **2009**; 200:888–99.

23. Orenstein WA, Cairns L, Hinman A, Nkowane B, Olive JM, Reingold AL. Measles and Rubella Global Strategic Plan 2012–2020 midterm review report: background and summary. Vaccine **2018**; 36:A35–42.

24. Patel MK, Gibson R, Cohen A, Dumolard L, Gacic-Dobo M. Global landscape of measles and rubella surveillance. Vaccine **2018**; 36:7385–92.

25. Bukasa A, Campbell H, Brown K, et al. Rubella infection in pregnancy and congenital rubella in United Kingdom, 2003 to 2016. Euro Surveill **2018**; 23:17-00381.

26. Seppälä EM, López-Perea N, Torres de Mier MV, Echevarría JE, Fernández-García A, Masa-Calles J. Last cases of rubella and congenital rubella syndrome in Spain, 1997-2016: the success of a vaccination program. Vaccine **2019**; 37:169–75.

27. Beraud G, Abrams S, Beutels P, Dervaux B, Hens N. Resurgence risk for measles, mumps and rubella in France in 2018 and 2020. Euro Surveill **2018**; 23:1700796.

28. Bianchi FP, De Nitto S, Stefanizzi P, Larocca AMV, Germinario CA, Tafuri S. Immunity to rubella: an Italian retrospective cohort study. BMC Public Health **2019**; 19:1490.

29. Giambi C, Filia A, Rota MC, et al. Congenital rubella still a public health problem in Italy: analysis of national surveillance data from 2005 to 2013. Euro Surveill **2015**; 20:21103.

30. Murhekar M, Bavdekar A, Benakappa A, et al. Sentinel surveillance for congenital rubella syndrome—India, 2016-2017. MMWR Morb Mortal Wkly Rep **2018**; 67:1012–6.

31. Pilania RK, Verma S, Kumar P, Sachdeva RK, Jayashree M, Singh M. Congenital rubella syndrome at tertiary care hospital in north India: results from a retrospective assessment. J Trop Pediatr **2019**; 65:297–300.

32. Wang C, Zhu Z, Xu Q, et al. Progress towards rubella elimination after implementation of rubella immunization for over 20 years in Shandong province, China. Sci Rep **2017**; 7:17982.

33. Meng Q, Luo J, Li L, et al. Rubella seroprevalence among pregnant women in Beijing, China. BMC Infect Dis **2018**; 18:130.

34. Su Q, Ma C, Wen N, et al. Epidemiological profile and progress toward rubella elimination in China. 10 years after nationwide introduction of rubella vaccine. Vaccine **2018**; 36:2079–85.

35. Lee H, Kayano T, Nishiura H. Predicting congenital rubella syndrome in Japan, 2018-2019. Int J Infect Dis **2019**; 82:1–5.

36. Jindai K, Funaki T, Nishijima T, Takakura S, Noda H, Miyake K. Towards rubella elimination in Japan. Lancet Infect Dis **2018**; 18:713–4.

37. Ujiie M. Rubella resurgence in Japan 2018–2019. J Travel Med **2019**; 26:taz047.

38. Edirisuriya C, Beard FH, Hendry AJ, et al. Australian rubella serosurvey 2012-2013: on track for elimination? Vaccine **2018**; 36:2794–8.

39. Durski KN, Tituli C, Ogaoga D, et al. An outbreak investigation of congenital rubella syndrome in Solomon Islands, 2013. Western Pac Surveill Response J **2016**; 7:10–3.

40. Pukuta E, Waku-Kouomou D, Abernathy E, et al. Genotypes of rubella virus and the epidemiology of rubella infections in the Democratic Republic of the Congo, 2004-2013. J Med Virol **2016**; 88:1677–84.

41. Kadjo HA, Waku-Kouomou D, Adagba M, et al. Epidemiology of rubella infection and genotyping of rubella virus in Cote d’Ivoire, 2012-2016. J Med Virol **2018**; 90:1687–94.

42. Dinede G, Wondimagegnehu A, Enquselassie F. Rubella outbreak in the school children, Addis Ababa, Ethiopia: February-April 2018. BMC Infect Dis **2019**; 19:267.

43. Gieles NC, Mutsaerts EAML, Kwatra G, et al. Rubella seroprevalence in pregnant women living with and without HIV in Soweto, South Africa. Int J Infect Dis **2020**; 91:255–60.

44. Mirambo MM, Aboud S, Mushi MF, et al. Serological evidence of acute rubella infection among under-fives in Mwanza: a threat to increasing rates of congenital rubella syndrome in Tanzania. Ital J Pediatr **2016**; 42:54.

45. Alfonso VH, Doshi RH, Mukadi P, et al. Prevalence of rubella antibodies among children in the Democratic Republic of the Congo. Pediatr Infect Dis J **2018**; 37:28–34.

46. Motaze NV, Manamela J, Smit S, et al. Congenital rubella syndrome surveillance in South Africa using a sentinel site approach: a cross-sectional study. Clin Infect Dis **2019**; 68:1658–64.

47. Mangtani P, Evans SJW, Lange B, et al. Safety profile of rubella vaccine administered to pregnant women: a systematic review of pregnancy related adverse events following immunisation, including congenital rubella syndrome and congenital rubella infection in the foetus or infant. Vaccine **2020**; 38:963–78.

48. Perelygina L, Chen MH, Suppiah S, et al. Infectious vaccinederived rubella viruses emerge, persist, and evolve in cutaneous granulomas of children with primary immunodeficiencies. PLoS Pathog **2019**; 15:e1008080.

49. Carryn S, Feyssaguet M, Povey M, Di Paolo E. Long-term immunogenicity of measles, mumps and rubella-containing vaccines in healthy young children: a 10-year follow-up. Vaccine **2019**; 37:5323–31.

50. Biellik R, Davidkin I, Esposito S, et al. Slow progress in finalizing measles and rubella elimination in the European Region. Health Aff (Millwood) **2016**; 35:322–6.

51. Hinman AR. Measles and rubella eradication. Vaccine **2018**; 36:1–3.
